# Religion and Voting Behavior of Older Adults With Disabilities in Ghana

**DOI:** 10.1093/geroni/igaa057.1280

**Published:** 2020-12-16

**Authors:** Kodwo-Nyameazea Yale

**Affiliations:** University of North Carolina at Pembroke, Pembroke, North Carolina, United States

## Abstract

Voting is a necessary and inherent right of citizens in democracies to select public office holders who decide how public goods and resources are distributed and maintained. It is therefore critical that all citizens who are eligible are able to participate in one of the key aspects of political participation – voting. This study focused on the factors that influence the ability of older adult Ghanaians with disabilities to vote in local and national elections. The study sample of 923 respondents was drawn from the second wave of WHO SAGE study on Global Aging and Adult Health. The results of the logistic regression analyses showed that religion influenced the voting behaviors of all the three people with disabilities groups included in the study. But certain groups are also influenced by interaction with community leaders and personal political interests and characteristics, including gender. Given these findings, it is suggested that an impact community be established around the meaning and ethics associated with the religious activities people with disabilities participate in, and engage them through civic engagement, and personal and community development activities that empower them to live meaningful lives.

